# High degree of sex chromosome differentiation in stickleback fishes

**DOI:** 10.1186/1471-2164-12-474

**Published:** 2011-09-29

**Authors:** Takahito Shikano, Heini M Natri, Yukinori Shimada, Juha Merilä

**Affiliations:** 1 Ecological Genetics Research Unit, Department of Biosciences, University of Helsinki, P.O. Box 65, FI-00014, Helsinki, Finland

## Abstract

**Background:**

Studies of closely related species with different sex chromosome systems can provide insights into the processes of sex chromosome differentiation and evolution. To investigate the potential utility of molecular markers in studying sex chromosome differentiation at early stages of their divergence, we examined the levels and patterns of genetic differentiation between sex chromosomes in nine-spined (*Pungitius pungitius*) and three-spined sticklebacks (*Gasterosteus aculeatus*) using microsatellite markers.

**Results:**

A set of novel microsatellite markers spanning the entire length of the sex chromosomes were developed for nine-spined sticklebacks using the sequenced genomes of other fish species. Sex-specific patterns of genetic variability and male-specific alleles were identified at most of these loci, indicating a high degree of differentiation between the X and Y chromosomes in nine-spined sticklebacks. In three-spined sticklebacks, male-specific alleles were detected at some loci confined to two chromosomal regions. In addition, male-specific null alleles were identified at several other loci, implying the absence of Y chromosomal alleles at these loci. Overall, male-specific alleles and null alleles were found over a region spanning 81% of the sex chromosomes in three-spined sticklebacks.

**Conclusions:**

High levels but distinct patterns of sex chromosome differentiation were uncovered in the stickleback species that diverged 13 million years ago. Our results suggest that the Y chromosome is highly degenerate in three-spined sticklebacks, but not in nine-spined sticklebacks. In general, the results demonstrate that microsatellites can be useful in identifying the degree and patterns of sex chromosome differentiation in species at initial stages of sex chromosome evolution.

## Background

Recent advances in comparative genomic and molecular cytogenetic studies have greatly increased our understanding about the extent and pace of sex chromosome evolution [1,2]. For instance, therian and avian sex chromosomes are known to have originated independently from two different autosomal regions in a common ancestor [1,3]. In contrast, lower vertebrates exhibit a wide variety of sex chromosome and sex determination systems, implying multiple and independent origins of sex chromosomes [4-6]. Recent studies have shown that sex chromosomes of *Oryzias *fishes and sticklebacks (Gasterosteidae) have emerged 10 million years ago or even later [7-10]. Likewise, different heterogametic sex determination systems have been found in tilapia fishes and the frog *Rana rugosa *[11,12].

Despite the substantial progress in understanding the evolutionary history of sex chromosomes, there are still gaps in our knowledge about the process of sex chromosome differentiation. For instance, it has been hypothesized that sex chromosomes would typically evolve from a pair of autosomes that cease to recombine with each other after acquiring a sex determining role [13-15]. Suppression of recombination leads to further differentiation of the sex chromosomes and degeneration of the heterogametic chromosome (i.e. Y or W), resulting in morphologically differentiated sex chromosomes [14,16]. Accordingly, a heteromorphic sex chromosome pair is generally thought to have evolved through increasing stages of differentiation [14]. However, since chromosomal rearrangements can produce heteromorphism, even newly evolved sex chromosomes can be heteromorphic [2]. In addition, neither suppressed recombination nor heteromorphism always evolve in old-established sex chromosomes [2].

Studies of model organisms with young sex chromosomes have uncovered molecular characteristics at the initial stages of differentiation that align with theoretical expectations [5,15,17]. Yet, molecular differentiation of sex chromosomes has been assessed mainly from sequence analyses, which are not easily accomplished without access to sequenced genomes or equivalent genomic resources [17]. Thus, simple and accurate methods of evaluating sex chromosome differentiation in non-model organisms would be desirable. One potential approach is to use allelic variation in genetic markers. Since allelic differentiation at microsatellite loci is expected to be in a linear relationship with time since divergence [18,19], microsatellite variation might be able to uncover the patterns and degree of sex chromosome differentiation. Indeed, although microsatellite markers have been rarely used for this purpose, they have been shown to be useful in detecting genetic differentiation between sex chromosomes in *Drosophila *[20]. Moreover, since a large proportion of microsatellite loci appear to be conserved in closely related species [21,22], the use of microsatellite markers may also allow comparative genomic analyses in this context. Conversely, the sequenced genomes of closely related species can be used to predict microsatellite locations in the genome of a target species lacking the reference genome sequence [22]. An obvious limitation in the development of markers is that mutations in the regions flanking microsatellite loci make it difficult to develop primers that work in a target species. Yet, this problem can be circumvented by designing primers in conserved regions, which are identifiable using the genome sequences of multiple species in the same taxon. Accordingly, based on microsatellite locations in the genome of a related species, microsatellite markers can be developed for target chromosomes and genomic regions in other species lacking sequenced genomes.

Sticklebacks are widely used as model organisms in evolutionary biology [23,24]. The genome sequence of the three-spined stickleback (*Gasterosteus aculeatus*) provides a useful resource for studying the genetic basis of several phenotypic traits [25]. A sex determining region has been mapped to linkage group (LG) 19 in three-spined sticklebacks - a species possessing an XY sex chromosome system [8]. Their sex chromosomes are cytologically indistinguishable in the absence of molecular cytogenetic information [26]. In contrast, nine-spined sticklebacks (*Pungitius pungitius*) have a heteromorphic XY pair corresponding to three-spined stickleback LG 12 [10,27]. In particular, their sex chromosomes are characterized by a large Y chromosome which might be a result of a tandem duplication of the ancient Y chromosome or a duplication of an autosomal segment followed by insertion into the Y chromosome [28]. These different sex chromosome systems suggest their independent evolution in stickleback species [10]. Because of the rapid turnover of sex chromosome systems in closely related species, sticklebacks provide an ideal system to study the initial stages of sex chromosome differentiation [8,10].

The aim of this study was to compare the level and patterns of genetic differentiation of sex chromosomes in nine-spined and three-spined sticklebacks using microsatellite markers. Since stickleback species exhibit a rapid turnover of sex chromosomes, their sex chromosomes are thought to be at the early stages of evolution. Therefore, we expected that the degree of genetic differentiation between sex chromosomes would be similar in nine-spined and three-spined sticklebacks. To facilitate the comparative genomic analyses, we devised a simple method for the development of microsatellite markers for target chromosomes and genomic regions in nine-spined sticklebacks using the sequenced genomes of other fish species.

## Results

### Nine-spined sticklebacks

**Figure 1:**
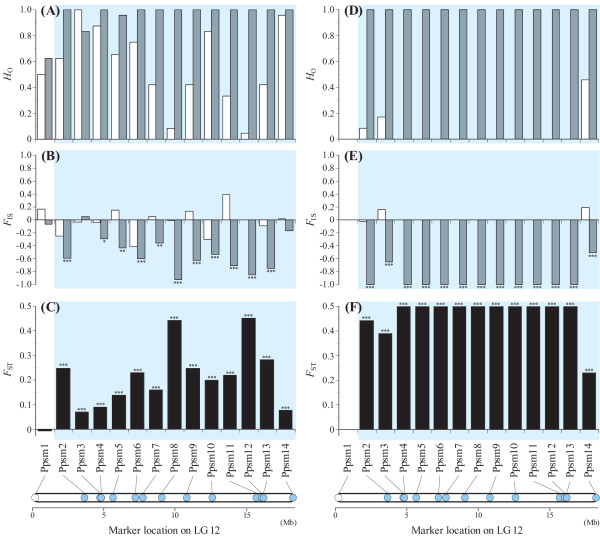
**Observed heterozygosity (*H*_O_), *F*_IS _and *F*_ST _between females and males at 14 Ppsm loci in nine-spined sticklebacks**. **A** and **D**, *H*_O _in females (white) and males (gray); **B **and **E**, *F*_IS _in females (white) and males (gray); **C **and **F**, *F*_ST _between females and males. **A**-**C**, Baltic Sea; **D**-**F**, Pyöreälampi. *P < 0.05, **P < 0.01 and ***P < 0.001. The blue colour indicates the loci where significant *F*_ST _was observed. Physical locations of loci were determined based on the three-spined stickleback genome.

Twenty-three polymorphic markers, including 14 for LG 12 (Ppsm), were developed for nine-spined sticklebacks (Table 1). On average, 10.6 alleles per locus (range = 2-47) were detected across the two populations (Table 1). The mean number of observed alleles and average expected heterozygosities were 10.3 and 0.66 in the Baltic Sea and 2.0 and 0.27 in Pyöreälampi, respectively (Additional file 1). Out of the 14 Ppsm loci, 10 were homozygous in all females and heterozygous in all males in the Pyöreälampi population (Figure 1, Additional file 1). Consequently, the number of observed alleles and expected and observed heterozygosities were significantly higher in males than females in this population (Wilcoxon signed rank test, *P *< 0.01 for allele number, *P *< 0.001 for heterozygosities). In the Baltic Sea population, observed heterozygosity was higher in males than females (*P *< 0.01; Figure 1), but no significant differences were found in the number of alleles and expected heterozygosity (*P *> 0.05). While no loci showed significant *F*_IS _in females, negative *F*_IS _was detected in males at 11 Ppsm loci in the Baltic Sea and at 13 Ppsm loci in Pyöreälampi (Figure 1, Additional file 1). Linkage disequilibrium was observed in all pairs of polymorphic Ppsm loci in Pyöreälampi, although 47 out of the 91 comparisons were not significant in the Baltic Sea (Additional file 2). In particular, linkage disequilibrium was not detected in several combinations including highly polymorphic loci (e.g. Ppsm3-5 and Ppsm14). The trend test identified significant associations between phenotypic sex and Ppsm loci in both populations (Additional file 3). All polymorphic Ppsm loci showed an association with sex in Pyöreälampi, whereas significant association was not observed for four Ppsm loci (Ppsm1, Ppsm3, Ppsm4 and Ppsm14) in the Baltic Sea (Additional file 3).

**Table 1:** Location of 23 microsatellites in the three-spined stickleback genome and primer sequences for nine-spined sticklebacks

Locus	Three-spined stickleback genome			Microsatellite marker for nine-spined stickleback		
		
	LG	Position (bp)	Repeat motif	Primer sequence (5'-3')	*A*	Allele size (bp)
Ppsm1	XII	188976	(CCT)_6_	F: GGCTCTTCCGATGAGTTCTC	6	298-308
				R: GTCTGCGCGTCAGCATCC		
Ppsm2	XII	3625645	(AC)_24_	F: GCCTCCCAGTCCTCTGT	5	175-183
				R: GCCATGGAGTACGACATCATG		
Ppsm3	XII	4802750	(GT)_16_	F: GAACGATGATTAATTTCACTC	42	161-271
				R: CTGACCCTGACTGGAGG		
Ppsm4	XII	4843632	(CA)_15_	F: CCAGCTGCTCTGTTTTGTTAAC	16	223-267
				R: CCTGGCCTCATTACAGTAAC		
Ppsm5	XII	5616109	(GAG)_7_	F: ATCACGACTCTGAGGAGAG	10	208-241
				R: TTCTTCAGCTCCACGGG		
Ppsm6	XII	7178391	(AC)_12_	F: ATCGCCCTGCTGGTGGAG	5	231-239
				R: GGAGCGCTGTTTCCGCC		
Ppsm7	XII	7695994	(GT)_13_	F: GCAGCACTGTTGTCCAA	9	73-143
				R: CTACTTGAACGATGCTC		
Ppsm8	XII	9015356	(GT)_15_	F: CCAAAGGCAATTTCAAATCTC	4	203-217
				R: GAATGACAGGCTGTTTGTCTG		
Ppsm9	XII	10780938	(TG)_16_	F: CAAGATGGACTACTCAAGG	3	247-252
				R: CTATCAACCTCTCCAGCTTC		
Ppsm10	XII	12581231	(TG)_7_	F: GCTTAGTGTTAATTGGTTCCTG	7	218-232
				R: TAGACCCTGAGGGTGTG		
Ppsm11	XII	15639505	(AT)_8_	F: AACAACGGTGCTATCTCCTCT	7	186-198
				R: TGGAATCCCATGCAGCGCAC		
Ppsm12	XII	16027500	(TG)_8_	F: GCATGGTCATCATCTGGAG	4	251-263
				R: ATGACACATGCATGGAGT		
Ppsm13	XII	16138995	(TG)_13_	F: GCCTGCTACAAAGCTGA	3	256-280
				R: CTTGAAGGACTCAAAGAAGCC		
Ppsm14	XII	18276705	(CA)_11_	F: CCGATGGCCTGGTTCAC	47	263-445
				R: GGCGTCATCATCACCGG		
Pprm1	III	4656850	(TG)_15_	F: TGTGCTGCAGACCTCCAC	5	202-214
				R: TGGCGTCACGGAGCTGAAG		
Pprm2	IV	62968	(CA)_13_	F: GATACAGCTCCTGCTCCAG	4	163-169
				R: CCAGGATGAACCAGGTGAG		
Pprm3	IV	15800213	(GT)_21_	F: GGCTTCTATTTCTGCCTCCC	23	344-392
				R: TACCTGAGCAGCTCGCAG		
Pprm4	V	4991466	(TGT)_6_	F: GCTGGGCAGTATTCTGTGG	7	288-306
				R: AACATCCTCATCCACAGCAGC		
Pprm5	XV	21776	(AC)_16_	F: ATCCCAACGTCATCCAGCTC	2	193-194
				R: CAGCAGGAAGGTGTGCAG		
Pprm6	XV	8323413	(CA)_13_	F: CTGGAGCGTTTACAGGTGG	8	364-382
				R: CTGCTGAGCTGAACAGGC		
Pprm7	XVI	3587216	(AC)_10_	F: CTGGAGACCAACAAGTTGAGG	12	274-298
				R: CTTAACAAAGATCCTGCTGGACG		
Pprm8	XVIII	10203501	(GT)_25_	F: CACCCATGTTCCTGTGCTTC	11	306-380
				R: ACAAAGCCCTGCTCTCGAG		
Pprm9	XX	5366535	(CA)_14_	F: TCCTCATGATGTTGACCAGTGC	3	166-172
				R: CTGGCCTATGGAAACCAGG		

LG, linkage group. F, forward; R, reverse. *A*, number of observed alleles.

The analyses of genotypic frequencies at Ppsm loci identified several alleles that were observed only in males (Table 2). All males from Pyöreälampi possessed both male-specific and nonspecific alleles at each of the polymorphic loci (Table 2). No polymorphism was detected in the male-specific alleles in this population. Male-specific alleles were found at nine loci in all males of the Baltic Sea (Table 2). Although a male-specific allele was not detected at Ppsm5, the same allele (229) was observed in most (23 out of 24) males and a few (2 out of 24) females. *F*_ST _values between females and males were significant for 13 Ppsm loci in both populations (Figure 1). For these loci, *F*_ST _values were larger in the Pyöreälampi than in the Baltic Sea.

**Table 2:** Male-specific alleles at 14 Ppsm loci in two populations of nine-spined sticklebacks

Locus	Baltic Sea		Pyöreälampi	
		
	Male-specific allele*	Other allele	Male-specific allele*	Other allele
Ppsm1	-	298-308	-	-
Ppsm2	179 (100.0)	175-181	179 (100.0)	181-183
Ppsm3	171 (54.2)	161-271	191 (100.0)	209-215
Ppsm4	247 (70.8)	223-267	247 (100.0)	227
Ppsm5	-	208-241	229 (100.0)	235
Ppsm6	232 (100.0)	231-239	232 (100.0)	235
Ppsm7	125-143 (100.0)	73-77	141 (100.0)	75
Ppsm8	203 (100.0)	211-217	203 (100.0)	215
Ppsm9	247 (100.0)	251-252	247 (100.0)	252
Ppsm10	222 (100.0)	218-232	222 (100.0)	220
Ppsm11	197 (100.0)	186-198	197 (100.0)	188
Ppsm12	251-253 (100.0)	257-263	253 (100.0)	257
Ppsm13	280 (100.0)	256-258	280 (100.0)	256
Ppsm14	287 (75.0)	263-445	279 (100.0)	281-287

*The percentage of males having the allele(s) is indicated in parentheses. Minor alleles (< 15%) are not shown except for Ppsm7 and Ppsm12.

### Three-spined sticklebacks

**Figure 2:**
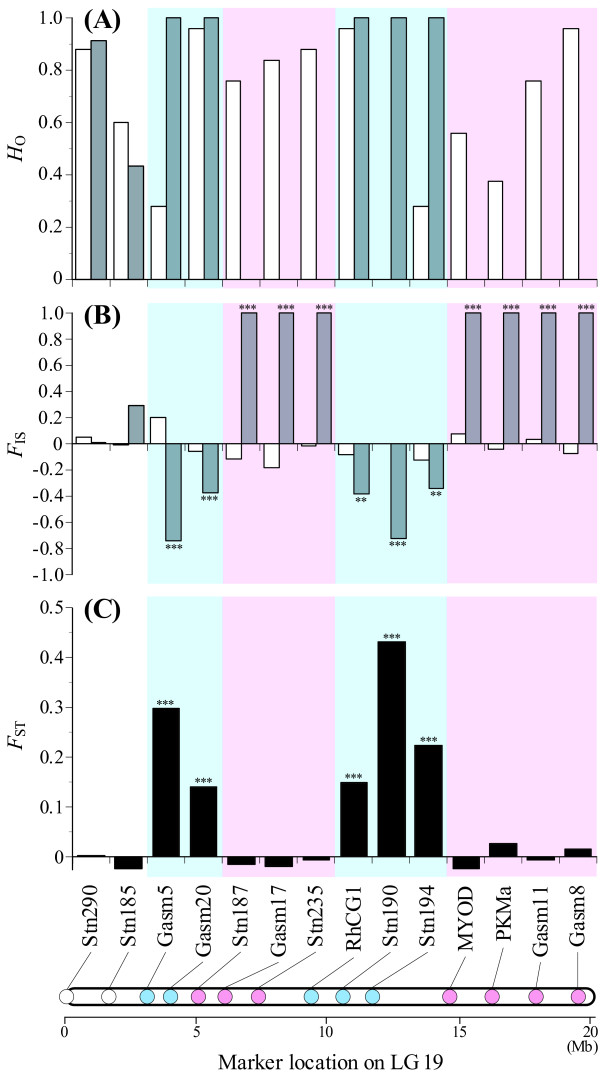
**Observed heterozygosity (*H*_O_), *F*_IS _and *F*_ST _between females and males at 14 loci in three-spined sticklebacks**. **A**, *H*_O _in females (white) and males (gray); **B**, *F*_IS _in females (white) and males (gray); **C**, *F*_ST _between females and males. **P < 0.01 and ***P < 0.001. The blue colour indicates the loci where significant *F*_ST _was observed, and the red colour represents the loci where no heterozygous males were found. Physical locations of loci were determined based on the three-spined stickleback genome but modified according to Ross and Peichel [26].

A total of 161 alleles were observed at 14 loci, with an average of 11.5 alleles per locus (range = 2-31) in three-spined sticklebacks (Additional files 4 and 5). Linkage disequilibrium was observed in only eight out of the 91 comparisons (Additional file 6). Expected heterozygosity ranged from 0.39 to 0.93 among the loci with a mean of 0.70 (Additional file 5). Several loci showed different levels of observed heterozygosity between females and males (Figure 2, Additional file 5). Although no loci showed significant *F*_IS _in females, 12 loci exhibited significant *F*_IS _in males (Figure 2, Additional file 5). Out of these 12 loci, five were heterozygous in all males, showing negative *F*_IS_. At these loci, which were located in two chromosomal regions (i.e. 3.2-4.0 Mb and 9.4-11.8 Mb), male-specific alleles were detected in all males in heterozygous states (Table 3). In contrast, although two to 20 alleles were observed for each locus, no heterozygous males were found at seven loci that showed positive *F*_IS _(Figure 2, Additional file 5). No male-specific alleles were identified at these loci (Table 3). MICRO-CHECKER analyses indicated the presence of null alleles at the seven loci in males, but not in females. These loci were located sequentially in two separate chromosomal regions (i.e. 5.1-7.4 Mb and 14.7-19.6 Mb) of LG 19 (Figure 2, Additional file 4).

**Table 3:** Male-specific alleles at 14 loci in three-spined sticklebacks

Locus	Baltic Sea	
	
	Male-specific allele*	Other allele
Stn290	-	100-178
Stn185	-	152-160
Gasm5	82 (100.0)	85-89
Gasm20	191 (100.0)	313-371
Stn187	-	156-184
Gasm17	-	193-213
Stn235	-	127-155
RhCG1	254 (100.0)	241-275
Stn190	231-279 (100.0)	183
Stn194	123-129 (100.0)	85-91
MYOD	-	150-162
PKMa	-	269-271
Gasm11	-	424-456
Gasm8	-	376-406

*The percentage of males having the allele(s) is indicated in parentheses. Minor alleles (< 15%) are not shown except for Stn194 and Stn190.

Significant associations between phenotypic sex and loci were detected for the five loci that possessed male-specific alleles (Additional file 7). *F*_ST _between females and males was significant for these, but not for the other loci (Figure 2).

## Discussion

Our study uncovered sex-specific patterns of genetic variability and male-specific alleles at several loci in both stickleback species. In addition, male-specific null alleles were identified at several loci in three-spined sticklebacks, suggesting the absence of Y chromosomal alleles at these loci. The high allelic heterogeneity between sexes indicates high levels of sex chromosome differentiation, which likely reflects recombination suppression between these chromosomes. In the following, we discuss these issues, as well as our approach to develop microsatellite markers in a non-model organism.

### Differentiation of stickleback sex chromosomes

Our results indicate that 13 markers (Ppsm2-14) are linked to each other, and are associated with phenotypic sex in nine-spined sticklebacks. These indications were more robust in a population with low genetic diversity (Pyöreälampi) than in one with high genetic diversity (Baltic Sea). Indeed, all males of Pyöreälampi possessed one particular male-specific allele for each of the 13 loci. Given the male heterogametic inheritance of this species [28], these alleles should be located on the Y chromosome. Accordingly, only one Y chromosome haplotype was identified in this population. In contrast, three out of the 13 loci did not exhibit an association with phenotypic sex in the Baltic Sea population. Since these three loci were highly polymorphic, this could be due to their high mutation rates. Even in this highly variable population, the male-specific allele was monomorphic at seven loci. However, polymorphisms were detected in the remaining alleles, which are putatively located on the X chromosome. The different level of polymorphism between the sex chromosomes is explainable by the fact that the effective population size of the Y chromosome is one-third of that of the X chromosome [29]. In general, our results indicate that sex chromosomes are highly differentiated throughout most of their length in nine-spined sticklebacks.

Based on karyotype analyses, the Y chromosome of nine-spined sticklebacks is characterized by a much larger size than the other chromosomes [10,28]. Because none of the autosomes or autosomal arms are missing, the additional segment observed in the Y chromosome is assumed to have originated from a tandem duplication of the ancient Y chromosome or a duplication of an autosomal segment followed by insertion into the Y chromosome [28]. Although we examined allelic variation along the sex chromosomes with several markers - for which primers were developed in conserved sequences of divergent species - no loci showed the patterns of multilocus amplification which can be an indication of the Y chromosome duplication. Thus, an extra segment of the Y chromosome is more likely to be derived from a duplication of an autosome than that of the Y chromosome.

In three-spined sticklebacks, an allozyme of isocitrate dehydrogenase (*IDH*) has been found to be associated with phenotypic sex [30,31]. In accordance with this observation, a linkage mapping analysis showed that the sex determining region is located near the *IDH *gene of LG 19 [8]. In our study, male-specific alleles, which should be located on the Y chromosome, were identified at five loci. Indeed, three of these loci were closely linked to the *IDH *gene (11.3 Mb). However, the remaining two loci were found in a chromosomal region (3.2-4.0 Mb) far from the *IDH *gene, implying that there is extensive differentiation. In addition, male-specific null alleles were identified at several loci located in two different chromosomal regions. This lack of heterozygous males suggests that amplification of Y chromosomal alleles is absent at these loci. This could be due to complete deletion of the microsatellite loci or polymorphisms in the primer binding sites of these loci in the Y chromosome. Overall, Y specific alleles and null alleles were identified over 16.4 Mb of a chromosomal region (3.2-19.6 Mb), which corresponds to 81% of the X chromosome. A large deletion of the Y chromosome was also suggested by a previous study using fluorescence *in situ *hybridization [26]. Indeed, the deletion was assumed to be present in one of the regions where Y chromosomal null alleles were identified (i.e. 14.7-19.6 Mb). Other Y chromosomal null alleles were found in the region where multiple inversions appear to have occurred [26]. It has also been demonstrated by sequence analysis that the Y chromosome exhibits substantial nucleotide divergence from the homologous region on the X chromosome mainly due to multiple insertions and deletions [8]. Together with these studies, our results suggest that the Y chromosome is highly degenerate in three-spined sticklebacks. Since closely related stickleback species exhibit the rapid turnover of sex chromosomes and sex determination systems, their sex chromosomes are thought to be at the early stages of evolution, as is generally observed in lower vertebrates [8,10]. Our study uncovered high levels but distinct patterns of sex chromosome differentiation in closely related stickleback species, which diverged about 13 million years ago [32]. In contrast to three-spined sticklebacks, we did not detect signatures of Y chromosome degeneration in the nine-spined stickleback despite the apparent sex chromosome heteromorphy in this species [28]. Therefore, it is unlikely that the Y chromosome of the nine-spined stickleback has degenerated as much as that of the three-spined stickleback.

Sexually antagonistic selection is expected to facilitate the suppression of recombination between sex chromosomes, and thus assumed to be the primary driving force behind sex chromosome differentiation [14]. Additionally, chromosome rearrangements, such as inversions, translocations and centric fusions involving an autosome and a sex chromosome, are known to reduce or suppress crossing over in the regions around the breakpoints in heterozygotes with the standard arrangement [14]. Such rearrangements can create linkage between sexually antagonistic genes and sex chromosomes, and thereby can be favored by natural selection [14,33]. As such, the high genetic divergence between the sex chromosomes of nine-spined and three-spined sticklebacks could be a result of both sexually antagonistic selection and chromosome rearrangements. Further research needs to be focused on the role of sexually antagonistic selection in sex chromosome differentiation in these species. In addition, it should be interesting to investigate intraspecific variation of sex chromosome differentiation and rearrangements among genetically divergent populations.

### Marker development for non-model organisms

To facilitate comparative genomic analyses, we devised a simple method for the development of microsatellite markers at target chromosomes and genomic regions using the sequenced genomes of other species. Our approach is similar to cross-species transfer of microsatellite markers, which can be performed without sequence information from a target species, but is different in the sense that conserved microsatellite flanking regions in divergent species are used as primer binding sites. Thus, it is possible to minimize amplification failures, which are a general concern in the cross-species utility of microsatellite markers [34]. In fact, when primers are designed using three-spined stickleback sequences alone, amplification success in nine-spined sticklebacks is low (28.6%) [22]. Moreover, our approach has an advantage over traditional cross-species amplification approaches because microsatellite markers can be developed for specific chromosomes and genomic regions based on the sequenced genome of other species. Furthermore, since primers are developed for conserved sequences in divergent species, these markers should be useful also in other stickleback species. Therefore, our approach for the development of microsatellite markers can facilitate comparative genomic analyses of stickleback species. In light of the rapidly increasing numbers of sequenced genomes and genomic resources, molecular tools for species lacking sequenced genomes can be developed using available genomic information of other species, as demonstrated in this study.

## Conclusions

To summarize, we uncovered high levels of sex chromosome differentiation in two stickleback species, suggesting that sex chromosomes have rapidly differentiated from each other. Our results further imply that the Y chromosome is highly degenerate in three-spined sticklebacks, but not in nine-spined sticklebacks. In general, our study demonstrates that microsatellites can be useful in identifying the degree and patterns of sex chromosome differentiation. Further comparative genomic analyses within and between stickleback species - possibly with the aid of the approach for the development of molecular markers described in this study - should facilitate our understanding of the evolutionary mechanisms underlying sex chromosome differentiation.

## Methods

### Fish samples

Mature nine-spined sticklebacks were collected from the Baltic Sea (60°12' N, 25°11' E) and from Pyöreälampi pond (66°16' N, 29°26' E) in 2008. These populations were selected to cover different levels of genetic diversity (average heterozygosity at 12 microsatellite loci, *H*_E _= 0.590 in Baltic Sea and *H*_E _= 0.004 in Pyöreälampi) [35], which can influence the identification of sex chromosome differentiation using microsatellite markers. Three-spined sticklebacks were sampled from the Baltic Sea (60°12' N, 25°11' E) in 2008. Phenotypic sex was determined by examining gonads after the fish had been anesthetised with an overdose of MS-222 (tricane methanesulphonate). All procedures were performed under license from the Animal Experiment Board in Finland (ELLA; STH379A).

### Microsatellite primer development

Using the three-spined stickleback genome [36], microsatellites were surveyed in regions randomly chosen over a wide range of LG 12 (Table 1), which corresponds to the sex chromosomes in nine-spined sticklebacks [10,27]. To design primers for nine-spined sticklebacks, conserved regions were searched in microsatellite flanking regions by aligning sequences of the three-spined stickleback and medaka [37]. Based on the location of microsatellites and conserved regions, primer sequences were designed manually, targeting appropriate melting temperature and GC content (Table 1). In addition to LG 12, some primers were developed for other linkage groups to verify variability of marker loci on sex chromosomes and autosomes (Table 1). For three-spined sticklebacks, 14 microsatellites covering a wide range of the sex chromosomes of this species (LG 19) were selected based on the sequenced genome [36] (Additional file 4). Of these, nine markers were previously reported [38,39], and new primers were developed for the remaining five loci with the genome sequences using WebSat [40]. Since the genome sequences were obtained from a female specimen [25], the novel primers were assumed to be designed based on the X chromosome sequences. All novel microsatellite primers were deposited in the National Center for Biotechnology Information (NCBI) Probe Database [PUIDs: 10552794-10552816, 10701232-10701236].

### Microsatellite genotyping

Total DNA was extracted from fin clips using a silica-fine based purification [41] following proteinase K digestion. Each forward primer was labelled with a fluorescent dye (FAM, HEX or TET), and the 5'-end of each reverse primer was modified with a GTTT-tail [42]. For efficient screening, PCRs were conducted using the Qiagen Multiplex PCR Kit (Qiagen) in 10 μl reaction volumes containing 1× Multiplex PCR Master Mix, 0.5× Q-Solution, 2 pmol of each primer and 10-20 ng of template DNA. The reactions were performed by the following cycle: an initial activation step at 95°C for 15 min, followed by 30 s at 94°C, 90 s at 53°C and 60 s at 72°C for 30 cycles with a final extension at 60°C for 5 min. PCR products were visualized with a MegaBACE 1000 automated sequencer (Amersham Biosciences) and their sizes were determined with ET-ROX 550 size standard (Amersham Biosciences). Polymorphism was initially screened using 24 individuals (12 females and 12 males) of nine-spined or three-spined sticklebacks from the Baltic Sea. For the identified polymorphic loci, a total of 48 individuals (24 females and 24 males for nine-spined sticklebacks and 25 females and 23 males for three-spined sticklebacks) were genotyped in each population. Alleles were scored using Fragment Profiler 1.2 (Amersham Biosciences) with visual inspection and manual corrections of alleles.

### Data analyses

Locus specific heterozygosity and *F*_IS _were calculated using FSTAT 2.9.3 [43]. The significance of *F*_IS _was assessed by 10 000 permutations. For marker loci located on sex chromosomes, *F*_IS _is expected to be lower in heterogametic males than in homogametic females if alleles are not shared between the sex chromosomes. Linkage disequilibrium was tested between pairs of loci in each population. An association between phenotypic sex and alleles at marker loci was investigated using the trend test [44,45] as implemented in PowerMarker 3.25 [46]. To identify Y chromosome specific alleles, genotypic frequencies and allele distributions were compared between males and females. In addition, to evaluate genetic differentiation between sex chromosomes, *F*_ST _was estimated between females and males using the method of Weir and Cockerham [47] as implemented in GENEPOP 4.0 [48]. Statistical significance of *F*_ST _values was determined using 10 000 permutations. In three-spined sticklebacks, some polymorphic loci appeared to be homozygous in all males (see Results). For these loci, the presence of sex-specific null alleles was tested using MICRO-CHECKER [49]. Since a large proportion of microsatellites are conserved between three-spined and nine-spined sticklebacks [22,27], microsatellite locations on LG 12 in nine-spined sticklebacks were estimated based on the three-spined stickleback genome. Based on the fact that the orientation of supercontig 3 of LG 19 in Ensembl is inverted as compared to the genetic map [26], marker locations on LG 19 were determined by reversing the sequence of this supercontig according to Ross and Peichel [26] (Additional file 4). Sequential Bonferroni corrections [50] were applied for all multiple comparisons to minimize type I errors.

## Authors' contributions

TS conceived of the study, performed the molecular work, conducted the data analyses and wrote the manuscript. HMN participated in the molecular work. YS collected the fish samples and assisted with the laboratory work. JM contributed to writing the manuscript. All authors read and approved the final manuscript.

## Supplementary Material

Additional file 1 **Number of observed alleles (*A*), observed and expected heterozygosities (*H*_O _and *H*_E_) and *F*_IS _at 23 loci in two populations of nine-spined sticklebacks**. Click here for file

Additional file 2 **Significance of linkage disequilibrium among 23 loci in the Baltic Sea (below the diagonal) and Pyöreälampi (above the diagonal) of nine-spined sticklebacks**. Click here for file

Additional file 3 **Association between phenotypic sex and loci in two populations of nine-spined sticklebacks**. Click here for file

Additional file 4 **Location of 14 microsatellites and primer sequences for three-spined sticklebacks**. Click here for file

Additional file 5 **Number of observed alleles (*A*), observed and expected heterozygosities (*H*_O _and *H*_E_) and *F*_IS _at 14 loci in three-spined sticklebacks**. Click here for file

Additional file 6 **Significance of linkage disequilibrium among 14 loci in three-spined sticklebacks**. Click here for file

Additional file 7 **Association between phenotypic sex and loci in three-spined sticklebacks**. Click here for file
