# Supplementary material for: High degree of sex chromosome differentiation in stickleback fishes
Source: BMC Genomics. 2011 Sep 29;12:474. doi: 10.1186/1471-2164-12-474 (PMC3201943; doi:10.1186/1471-2164-12-474)
Supplement: Additional file 1 — Number of observed alleles (A), observed and expected heterozygosities (HO and HE) and FIS at 23 loci in two populations of nine-spined sticklebacks. [file 1471-2164-12-474-S1.PDF]

Additional file 1 Number of observed alleles (*A*), observed and expected heterozygosities (H_O and H_E) and F_{IS} at 23 loci in two populations of nine-spined sticklebacks

Locus	Baltic Sea												Pyöreälampi											
	All				Female				Male				All				Female				Male			
	A	H_O	H_E	F_{IS}	*A*	H_O	H_E	F_{IS}	*A*	H_O	H_E	F_{IS}	*A*	H_O	H_E	F_{IS}	*A*	H_O	H_E	F_{IS}	*A*	H_O	H_E	F_{IS}
Ppsm1	6	0.563	0.594	0.053	3	0.500	0.602	0.170	6	0.625	0.589	-0.062	1	0.000	0.000	na	1	0.000	0.000	na	1	0.000	0.000	na
Ppsm2	4	0.813	0.660	-0.231	2	0.625	0.500	-0.250	4	1.000	0.630	-0.588***	3	0.542	0.408	-0.327	2	0.083	0.082	-0.022	2	1.000	0.500	-1.000***
Ppsm3	40	0.911	0.956	0.047	26	1.000	0.973	-0.028	22	0.833	0.875	0.048	5	0.583	0.533	-0.093	4	0.167	0.197	0.156	5	1.000	0.608	-0.645***
Ppsm4	15	0.938	0.849	-0.105	13	0.875	0.839	-0.043	8	1.000	0.777	-0.287*	2	0.500	0.378	-0.324	1	0.000	0.000	na	2	1.000	0.500	-1.000***
Ppsm5	10	0.809	0.778	-0.040	8	0.652	0.768	0.151	7	0.958	0.671	-0.428**	2	0.500	0.378	-0.324	1	0.000	0.000	na	2	1.000	0.500	-1.000***
Ppsm6	5	0.875	0.668	-0.309*	4	0.750	0.533	-0.408	3	1.000	0.627	-0.595***	2	0.500	0.378	-0.324	1	0.000	0.000	na	2	1.000	0.500	-1.000***
Ppsm7	8	0.696	0.641	-0.086	3	0.417	0.441	0.055	7	1.000	0.739	-0.353**	2	0.500	0.378	-0.324	1	0.000	0.000	na	2	1.000	0.500	-1.000***
Ppsm8	4	0.542	0.424	-0.279	3	0.083	0.082	-0.011	3	1.000	0.521	-0.920***	2	0.500	0.378	-0.324	1	0.000	0.000	na	2	1.000	0.500	-1.000***
Ppsm9	3	0.708	0.641	-0.106	2	0.417	0.480	0.132	3	1.000	0.616	-0.624***	2	0.500	0.378	-0.324	1	0.000	0.000	na	2	1.000	0.500	-1.000***
Ppsm10	7	0.917	0.730	-0.256*	5	0.833	0.642	-0.298	5	1.000	0.653	-0.531***	2	0.500	0.378	-0.324	1	0.000	0.000	na	2	1.000	0.500	-1.000***
Ppsm11	7	0.667	0.650	-0.025	6	0.333	0.552	0.396	3	1.000	0.586	-0.706***	2	0.500	0.378	-0.324	1	0.000	0.000	na	2	1.000	0.500	-1.000***
Ppsm12	4	0.521	0.414	-0.259	2	0.042	0.042	0.000	3	1.000	0.541	-0.849***	2	0.500	0.378	-0.324	1	0.000	0.000	na	2	1.000	0.500	-1.000***
Ppsm13	3	0.708	0.574	-0.234	2	0.417	0.382	-0.090	3	1.000	0.572	-0.747***	2	0.500	0.378	-0.324	1	0.000	0.000	na	2	1.000	0.500	-1.000***
Ppsm14	45	0.979	0.954	-0.026	30	0.958	0.973	0.015	26	1.000	0.856	-0.168	5	0.729	0.709	-0.029	3	0.458	0.565	0.189	5	1.000	0.665	-0.504***
Pprm1	5	0.542	0.545	0.006	5	0.583	0.599	0.026	4	0.500	0.490	-0.020	2	0.125	0.155	0.192	2	0.167	0.156	-0.070	2	0.083	0.158	0.471
Pprm2	4	0.438	0.363	-0.204	2	0.208	0.190	-0.095	4	0.667	0.505	-0.321	1	0.000	0.000	na	1	0.000	0.000	na	1	0.000	0.000	na
Pprm3	23	0.875	0.889	0.015	18	0.875	0.893	0.020	16	0.875	0.890	0.017	1	0.000	0.000	na	1	0.000	0.000	na	1	0.000	0.000	na
Pprm4	7	0.583	0.596	0.022	7	0.625	0.646	0.032	7	0.542	0.554	0.023	2	0.292	0.308	0.053	2	0.250	0.284	0.121	2	0.333	0.337	0.011
Pprm5	2	0.333	0.485	0.313	2	0.417	0.382	-0.090	2	0.250	0.513	0.512	1	0.000	0.000	na	1	0.000	0.000	na	1	0.000	0.000	na
Pprm6	8	0.833	0.723	-0.152	6	0.875	0.729	-0.200	6	0.792	0.713	-0.111	1	0.000	0.000	na	1	0.000	0.000	na	1	0.000	0.000	na
Pprm7	12	0.917	0.804	-0.140	11	0.958	0.841	-0.139	7	0.875	0.770	-0.136	2	0.521	0.388	-0.343	2	0.500	0.380	-0.314	2	0.542	0.400	-0.353
Pprm8	11	0.667	0.737	0.095	9	0.583	0.742	0.214	9	0.750	0.741	-0.012	1	0.000	0.000	na	1	0.000	0.000	na	1	0.000	0.000	na
Pprm9	3	0.438	0.488	0.104	3	0.417	0.566	0.264	3	0.458	0.399	-0.147	1	0.000	0.000	na	1	0.000	0.000	na	1	0.000	0.000	na

na, not applied. *$P < 0.05$, **$P < 0.01$, ***$P < 0.001$.
