# Supplementary material for: High degree of sex chromosome differentiation in stickleback fishes
Source: BMC Genomics. 2011 Sep 29;12:474. doi: 10.1186/1471-2164-12-474 (PMC3201943; doi:10.1186/1471-2164-12-474)
Supplement: Additional file 2 — Significance of linkage disequilibrium among 23 loci in the Baltic Sea (below the diagonal) and Pyöreälampi (above the diagonal) of nine-spined sticklebacks. [file 1471-2164-12-474-S2.PDF]

Additional file 2 Significance of linkage disequilibrium among 23 loci in the Baltic Sea (below the diagonal) and Pyöreälampi (above the diagonal) of nine-spined sticklebacks

	Ppsm1	Ppsm2	Ppsm3	Ppsm4	Ppsm5	Ppsm6	Ppsm7	Ppsm8	Ppsm9	Ppsm10	Ppsm11	Ppsm12	Ppsm13	Ppsm14	Pprm1	Pprm2	Pprm3	Pprm4	Pprm5	Pprm6	Pprm7	Pprm8	Pprm9
Ppsm1		na	na	na	na	na	na	na	na	na	na	na	na	na	na	na	na	na	na	na	na	na	na
Ppsm2	0.7859		**0.0000**	**0.0000**	**0.0000**	**0.0000**	**0.0000**	**0.0000**	**0.0000**	**0.0000**	**0.0000**	**0.0000**	**0.0000**	**0.0000**	0.3733	na	na	0.8102	na	na	1.0000	na	na
Ppsm3	0.6397	0.6656		**0.0000**	**0.0000**	**0.0000**	**0.0000**	**0.0000**	**0.0000**	**0.0000**	**0.0000**	**0.0000**	**0.0000**	**0.0000**	0.3935	na	na	0.7939	na	na	0.8228	na	na
Ppsm4	0.9178	0.0167	0.1984		**0.0000**	**0.0000**	**0.0000**	**0.0000**	**0.0000**	**0.0000**	**0.0000**	**0.0000**	**0.0000**	**0.0000**	0.6656	na	na	0.8735	na	na	0.7807	na	na
Ppsm5	0.6955	**0.0002**	0.0013	0.0052		**0.0000**	**0.0000**	**0.0000**	**0.0000**	**0.0000**	**0.0000**	**0.0000**	**0.0000**	**0.0000**	0.6659	na	na	0.8714	na	na	0.7849	na	na
Ppsm6	0.2729	**0.0000**	0.1835	0.0018	**0.0001**		**0.0000**	**0.0000**	**0.0000**	**0.0000**	**0.0000**	**0.0000**	**0.0000**	**0.0000**	0.6679	na	na	0.8722	na	na	0.7794	na	na
Ppsm7	0.0319	**0.0000**	0.4398	0.0159	0.0966	**0.0000**		**0.0000**	**0.0000**	**0.0000**	**0.0000**	**0.0000**	**0.0000**	**0.0000**	0.6694	na	na	0.7619	na	na	1.0000	na	na
Ppsm8	0.2113	**0.0000**	0.0817	**0.0000**	**0.0000**	**0.0000**	**0.0000**		**0.0000**	**0.0000**	**0.0000**	**0.0000**	**0.0000**	**0.0000**	0.6649	na	na	0.8736	na	na	0.7795	na	na
Ppsm9	0.7937	**0.0000**	0.1609	**0.0000**	**0.0000**	**0.0000**	**0.0000**	**0.0000**		**0.0000**	**0.0000**	**0.0000**	**0.0000**	**0.0000**	0.6685	na	na	0.8729	na	na	0.7831	na	na
Ppsm10	0.4323	**0.0000**	0.0333	0.0070	**0.0001**	**0.0000**	0.0007	**0.0000**	**0.0000**		**0.0000**	**0.0000**	**0.0000**	**0.0000**	0.6659	na	na	0.7666	na	na	1.0000	na	na
Ppsm11	0.2545	**0.0000**	0.1687	0.0877	**0.0000**	**0.0000**	**0.0000**	**0.0000**	**0.0000**	**0.0000**		**0.0000**	**0.0000**	**0.0000**	0.6671	na	na	0.7678	na	na	1.0000	na	na
Ppsm12	0.5749	**0.0000**	0.0821	0.0070	**0.0000**	**0.0000**	**0.0000**	**0.0000**	**0.0000**	**0.0000**	**0.0000**		**0.0000**	**0.0000**	0.6631	na	na	0.8725	na	na	0.7806	na	na
Ppsm13	0.2353	**0.0000**	0.2513	0.0033	0.0007	**0.0000**	**0.0000**	**0.0000**	**0.0000**	**0.0000**	**0.0000**	**0.0000**		**0.0000**	0.6663	na	na	0.7645	na	na	1.0000	na	na
Ppsm14	1.0000	1.0000	1.0000	1.0000	1.0000	1.0000	1.0000	1.0000	1.0000	1.0000	1.0000	1.0000	1.0000		0.3118	na	na	0.4399	na	na	0.0011	na	na
Pprm1	0.3784	0.1187	0.6573	0.1599	0.0298	0.0135	0.2983	0.0905	0.1903	0.0047	0.1297	0.0401	0.1075	1.0000		na	na	0.1862	na	na	0.6732	na	na
Pprm2	0.1106	0.0707	0.4764	0.3940	0.4476	0.2650	0.0040	0.0515	0.0087	0.1213	0.0614	0.0330	0.0002	1.0000	0.1446		na	na	na	na	na	na	na
Pprm3	0.4201	0.0486	0.0598	0.6079	0.8332	0.3494	0.3557	0.7797	0.9269	0.7907	0.3124	0.5547	0.5237	1.0000	0.9337	0.0025		na	na	na	na	na	na
Pprm4	0.4600	0.8911	0.0639	0.3081	0.3675	0.6635	0.9469	0.7788	0.4490	0.0471	0.7338	0.8810	0.5048	1.0000	0.9042	0.6878	0.4593		na	na	0.5947	na	na
Pprm5	0.7928	0.2296	0.8593	0.8162	0.4054	0.0085	0.0153	0.0134	0.0129	0.1146	0.0069	0.0052	0.0161	1.0000	0.0001	0.3489	0.6512	0.0134		na	na	na	na
Pprm6	0.5156	0.9167	0.0953	0.1174	0.5075	0.2044	0.5568	0.1176	0.2083	0.5029	0.2459	0.8080	0.6430	1.0000	0.7403	0.2492	0.2658	0.2733	0.9327		na	na	na
Pprm7	0.1447	0.4028	1.0000	0.0177	0.5756	0.3492	0.0575	0.6710	0.5552	0.2368	0.3200	0.8783	0.2008	1.0000	0.6615	0.8404	1.0000	0.6148	0.9589	0.0427		na	na
Pprm8	0.8114	0.7048	1.0000	0.3339	0.1455	0.7361	0.3080	0.6367	0.8110	0.6349	0.8729	0.7797	0.9813	1.0000	0.2806	0.9582	1.0000	0.4615	0.3929	0.4703	0.9488		na
Pprm9	0.4347	0.4374	0.8291	0.3375	0.0241	0.5991	0.3276	0.0179	0.0017	0.0865	0.0502	0.5013	0.8663	1.0000	0.1906	0.7845	0.8444	0.7760	0.0110	0.4610	0.5927	0.6077	

na, not applied. Bold, $P < 0.05$.
