# Supplementary material for: High degree of sex chromosome differentiation in stickleback fishes
Source: BMC Genomics. 2011 Sep 29;12:474. doi: 10.1186/1471-2164-12-474 (PMC3201943; doi:10.1186/1471-2164-12-474)
Supplement: Additional file 3 — Association between phenotypic sex and loci in two populations of nine-spined sticklebacks. [file 1471-2164-12-474-S3.PDF]

Additional file 3 Association between phenotypic sex and loci in two populations of nine-spined sticklebacks

Locus	Baltic Sea			Pyöreälampi		
	χ^2	d.f.	*P*	χ^2	d.f.	*P*
Ppsm1	4.4	5	0.499	-	-	-
Ppsm2	48.0	3	2.1×10^{-10}***	48.0	2	3.8×10^{-11}***
Ppsm3	44.4	39	0.256	48.0	4	9.4×10^{-10}***
Ppsm4	33.8	14	0.002	48.0	1	4.3×10^{-12}***
Ppsm5	37.9	9	1.8×10^{-5}***	48.0	1	4.3×10^{-12}***
Ppsm6	48.0	4	9.4×10^{-10}***	48.0	1	4.3×10^{-12}***
Ppsm7	46.0	7	8.7×10^{-8}***	48.0	1	4.3×10^{-12}***
Ppsm8	48.0	3	2.1×10^{-10}***	48.0	1	4.3×10^{-12}***
Ppsm9	48.0	2	3.8×10^{-11}***	48.0	1	4.3×10^{-12}***
Ppsm10	48.0	6	1.2×10^{-8}***	48.0	1	4.3×10^{-12}***
Ppsm11	48.0	6	1.2×10^{-8}***	48.0	1	4.3×10^{-12}***
Ppsm12	46.1	3	5.4×10^{-10}***	48.0	1	4.3×10^{-12}***
Ppsm13	48.0	2	3.8×10^{-11}***	48.0	1	4.3×10^{-12}***
Ppsm14	25.0	44	0.991	48.0	4	9.4×10^{-10}***
Pprm1	7.2	4	0.126	0.0	1	1.000
Pprm2	11.7	3	0.008	-	-	-
Pprm3	17.0	22	0.763	-	-	-
Pprm4	2.6	6	0.863	0.3	1	0.609
Pprm5	6.6	1	0.010	-	-	-
Pprm6	8.3	7	0.307	-	-	-
Pprm7	10.0	11	0.534	0.1	1	0.773
Pprm8	6.5	10	0.771	-	-	-
Pprm9	2.7	2	0.264	-	-	-

***$P < 0.001$.
