# Supplementary material for: High degree of sex chromosome differentiation in stickleback fishes
Source: BMC Genomics. 2011 Sep 29;12:474. doi: 10.1186/1471-2164-12-474 (PMC3201943; doi:10.1186/1471-2164-12-474)
Supplement: Additional file 4 — Location of 14 microsatellites and primer sequences for three-spined sticklebacks. [file 1471-2164-12-474-S4.PDF]

Additional file 4 Location of 14 microsatellites and primer sequences for three-spined sticklebacks

Locus	Three-spined stickleback genome				Microsatellite marker for nine-spined stickleback		
	LG	Position (bp)	Modified position (bp)*	Repeat motif	Primer sequence (5'-3') or reference	*A*	Allele size (bp)
Stn290	XIX	20116	20116	$(AC)_{20}$	GenBank accession no. BV678101	31	100-178
Stn185	XIX	1643381	1643381	$(TG)_{19}$	Peichel et al. [37]	5	152-160
Gasm5	XIX	3157439	3157439	$(AC)_{16}$	F: GTTGCAGGGAGACAAGTGAT<br>R: CCCGAGGACACAAAAGAAA	3	82-89
Gasm20	XIX	20047231	4013682	$(GT)_{13}$	F: ACATGGTGTTTGGAGGGAG<br>R: ACTGAGAAGGAGGCAGAGGT	22	191-371
Stn187	XIX	18969871	5091042	$(CA)_{15}$	Peichel et al. [37]	10	156-184
Gasm17	XIX	17947339	6113574	$(CA)_{21}$	F: CAAAGGTGGGAACTAAACTCTC<br>R: GGGCTGACTGTTAAAGATGG	8	193-213
Stn235	XIX	16667313	7393600	$(TG)_{16}$	GenBank accession no. BV678166	13	127-155
RhCG1	XIX	14617968	9442945	$(TC)_{14}$	Shimada et al. [38]	16	241-275
Stn190	XIX	13403011	10657902	$(TG)_{7}$	Peichel et al. [37]	5	183-279
Stn194	XIX	12275379	11785534	$(GT)_{18}$	Peichel et al. [37]	8	85-129
MYOD	XIX	9372452	14688461	$(GAG)_{4}$	Shimada et al. [38]	5	150-162
PKMa	XIX	7694202	16366711	$(AC)_{8}$	Shimada et al. [38]	2	269-271
Gasm11	XIX	6087120	17973793	$(AC)_{16}$	F: TGAACTGTCGTGTCGGTATG<br>R: GGGGATTCTTGTGCTGAAAT	13	424-456
Gasm8	XIX	4471738	19589175	$(GT)_{32}$	F: TGCAGACAAGCTGACTTTTG<br>R: CTAGAAGACCCCATCCTGCT	20	376-406

LG, linkage group. F, forward; R, reverse. *A* , number of observed alleles. * modified according to Ross and Peichel [26].
