# Supplementary material for: High degree of sex chromosome differentiation in stickleback fishes
Source: BMC Genomics. 2011 Sep 29;12:474. doi: 10.1186/1471-2164-12-474 (PMC3201943; doi:10.1186/1471-2164-12-474)
Supplement: Additional file 5 — Number of observed alleles (A), observed and expected heterozygosities (HO and HE) and FIS at 14 loci in three-spined sticklebacks. [file 1471-2164-12-474-S5.PDF]

**Additional file 5 Number of observed alleles (*A*), observed and expected heterozygosities ($H_O$ and $H_E$) and $F_{IS}$ at 14 loci in three-spined sticklebacks**

| Locus | Baltic Sea | | | | | | | | | | | |
|---|---|---|---|---|---|---|---|---|---|---|---|---|
| | All | | | | Female | | | | Male | | | |
| | $A$ | $H_O$ | $H_E$ | $F_{IS}$ | $A$ | $H_O$ | $H_E$ | $F_{IS}$ | $A$ | $H_O$ | $H_E$ | $F_{IS}$ |
| Stn290 | 31 | 0.896 | 0.926 | 0.033 | 21 | 0.880 | 0.927 | 0.050 | 22 | 0.913 | 0.924 | 0.012 |
| Stn185 | 5 | 0.521 | 0.598 | 0.129 | 5 | 0.600 | 0.597 | -0.006 | 5 | 0.435 | 0.615 | 0.293 |
| Gasm5 | 3 | 0.625 | 0.558 | -0.119 | 2 | 0.280 | 0.352 | 0.204 | 3 | 1.000 | 0.575 | -0.739*** |
| Gasm20 | 22 | 0.979 | 0.888 | -0.103 | 17 | 0.958 | 0.908 | -0.056 | 14 | 1.000 | 0.727 | -0.375*** |
| Stn187 | 10 | 0.396 | 0.694 | 0.430*** | 9 | 0.760 | 0.683 | -0.114 | 6 | 0.000 | 0.719 | 1.000*** |
| Gasm17 | 8 | 0.438 | 0.676 | 0.353*** | 8 | 0.840 | 0.710 | -0.183 | 5 | 0.000 | 0.652 | 1.000*** |
| Stn235 | 13 | 0.458 | 0.838 | 0.453*** | 12 | 0.880 | 0.865 | -0.017 | 10 | 0.000 | 0.814 | 1.000*** |
| RhCG1 | 16 | 0.979 | 0.883 | -0.109 | 12 | 0.960 | 0.887 | -0.083 | 9 | 1.000 | 0.722 | -0.385** |
| Stn190 | 5 | 0.479 | 0.385 | -0.245 | 1 | 0.000 | 0.000 | na | 5 | 1.000 | 0.580 | -0.724*** |
| Stn194 | 8 | 0.625 | 0.560 | -0.116 | 3 | 0.280 | 0.250 | -0.120 | 8 | 1.000 | 0.748 | -0.337** |
| MYOD | 5 | 0.292 | 0.632 | 0.539*** | 5 | 0.560 | 0.607 | 0.077 | 4 | 0.000 | 0.676 | 1.000*** |
| PKMa | 2 | 0.191 | 0.434 | 0.559*** | 2 | 0.375 | 0.361 | -0.040 | 2 | 0.000 | 0.498 | 1.000*** |
| Gasm11 | 13 | 0.396 | 0.803 | 0.507*** | 11 | 0.760 | 0.790 | 0.038 | 7 | 0.000 | 0.822 | 1.000*** |
| Gasm8 | 20 | 0.500 | 0.894 | 0.440*** | 18 | 0.960 | 0.892 | -0.077 | 10 | 0.000 | 0.881 | 1.000*** |

na, not applied. $^{**}P < 0.01$, $^{***}P < 0.001$.
