# Supplementary material for: High degree of sex chromosome differentiation in stickleback fishes
Source: BMC Genomics. 2011 Sep 29;12:474. doi: 10.1186/1471-2164-12-474 (PMC3201943; doi:10.1186/1471-2164-12-474)
Supplement: Additional file 6 — Significance of linkage disequilibrium among 14 loci in three-spined sticklebacks. [file 1471-2164-12-474-S6.PDF]

Additional file 6 Significance of linkage disequilibrium among 14 loci in three-spined sticklebacks

	Stn290	Stn185	Gasm5	Gasm20	Stn187	Gasm17	Stn235	RhCG1	Stn190	Stn194	MYOD	PKMa	Gasm11	Gasm8
Stn290														
Stn185	0.1929													
Gasm5	0.5766	0.2947												
Gasm20	1.0000	0.8980	0.0010											
Stn187	0.8267	0.3917	0.0033	0.2615										
Gasm17	0.8594	0.1766	**0.0001**	0.0060	0.0104									
Stn235	1.0000	0.6322	0.0440	0.4789	0.0514	0.0125								
RhCG1	1.0000	0.3437	**0.0000**	0.1872	0.1267	0.1126	0.2111							
Stn190	0.6403	0.7933	**0.0000**	0.0006	0.0020	**0.0001**	0.0344	**0.0000**						
Stn194	0.6410	0.8675	**0.0000**	0.4952	0.4246	0.2715	0.0388	0.0146	**0.0000**					
MYOD	0.7456	0.5364	0.0100	0.4958	0.0886	0.3073	0.4822	0.0131	0.0178	0.1773				
PKMa	0.7108	0.8090	0.0015	0.0420	0.4229	0.2251	0.2253	0.0754	0.0022	0.0851	0.0705			
Gasm11	0.6267	0.8876	0.0086	0.1583	0.0977	0.0013	0.4499	0.9746	0.0305	0.1707	0.1154	0.0767		
Gasm8	1.0000	0.6644	0.0018	0.0166	0.1091	0.0033	0.0355	**0.0003**	0.0023	0.2696	0.1945	0.0729	0.0329	

Bold, $P < 0.05$.
