# Supplementary material for: High degree of sex chromosome differentiation in stickleback fishes
Source: BMC Genomics. 2011 Sep 29;12:474. doi: 10.1186/1471-2164-12-474 (PMC3201943; doi:10.1186/1471-2164-12-474)
Supplement: Additional file 7 — Association between phenotypic sex and loci in three-spined sticklebacks. [file 1471-2164-12-474-S7.PDF]

Additional file 7 Association between phenotypic sex and loci in three-spined sticklebacks

Locus	Baltic Sea		
	χ^2	d.f.	*P*
Stn290	30.8	30	0.426
Stn185	0.1	4	1.000
Gasm5	48.0	2	3.8×10^{-11}***
Gasm20	47.0	21	9.4×10^{-4}*
Stn187	7.2	9	0.611
Gasm17	5.1	7	0.645
Stn235	12.0	12	0.449
RhCG1	47.0	15	3.7×10^{-5}***
Stn190	48.0	4	9.4×10^{-10}***
Stn194	48.0	7	3.6×10^{-8}***
MYOD	2.4	4	0.664
PKMa	1.9	1	0.172
Gasm11	14.0	12	0.300
Gasm8	23.1	19	0.232

* $P < 0.05$, ***$P < 0.001$.
